# Novel immune genes associated with excessive inflammatory and antiviral responses to rhinovirus in COPD

**DOI:** 10.1186/1465-9921-14-15

**Published:** 2013-02-06

**Authors:** Katherine J Baines, Alan C-Y Hsu, Melinda Tooze, Lakshitha P Gunawardhana, Peter G Gibson, Peter AB Wark

**Affiliations:** 1 Priority Research Centre for Asthma and Respiratory Diseases, The University of Newcastle, Callaghan, NSW, Australia; 2 Virus, Infections/Immunity, Vaccines, & Asthma, Hunter Medical Research Institute, Lot 1, Kookaburra Circuit, New Lambton Heights, NSW, Australia; 3 Department of Respiratory and Sleep Medicine, John Hunter Hospital, New Lambton Heights, NSW, Australia

**Keywords:** COPD, Immune response, Viral infection, Gene expression

## Abstract

**Background:**

Rhinovirus (RV) is a major cause of chronic obstructive pulmonary disease (COPD) exacerbations, and primarily infects bronchial epithelial cells. Immune responses from BECs to RV infection are critical in limiting viral replication, and remain unclear in COPD. The objective of this study is to investigate innate immune responses to RV infection in COPD primary BECs (pBECs) in comparison to healthy controls.

**Methods:**

Primary bronchial epithelial cells (pBECs) from subjects with COPD and healthy controls were infected with RV-1B. Cells and cell supernatant were collected and analysed using gene expression microarray, qPCR, ELISA, flow cytometry and titration assay for viral replication.

**Results:**

COPD pBECs responded to RV-1B infection with an increased expression of antiviral and pro-inflammatory genes compared to healthy pBECs, including cytokines, chemokines, RNA helicases, and interferons (IFNs). Similar levels of viral replication were observed in both disease groups; however COPD pBECs were highly susceptible to apoptosis. COPD pBECs differed at baseline in the expression of 9 genes, including calgranulins S100A8/A9, and 22 genes after RV-1B infection including the signalling proteins pellino-1 and interleukin-1 receptor associated kinase 2. In COPD, IFN-β/λ1 pre-treatment did not change MDA-5/RIG-I and IFN-β expression, but resulted in higher levels IFN-λ1, CXCL-10 and CCL-5. This led to reduced viral replication, but did not increase pro-inflammatory cytokines.

**Conclusions:**

COPD pBECs elicit an exaggerated pro-inflammatory and antiviral response to RV-1B infection, without changing viral replication. IFN pre-treatment reduced viral replication. This study identified novel genes and pathways involved in potentiating the inflammatory response to RV in COPD.

## Background

Chronic obstructive pulmonary disease (COPD) is responsible for an increasing burden of illness and death around the world. COPD is chronic airway disease, characterized by incompletely reversible airflow obstruction, and symptoms of cough and sputum production. These symptoms can be worsened with exposure to microbial infections [1]. Rhinoviruses (RVs) are the most frequently detected viruses during acute exacerbation [2], and infection is associated with rapid decline in lung function and severe symptoms that often requires hospitalization [2]. However the specific mechanism that leads to this enhanced susceptibility and severe symptoms following RV infection is not well understood.

Bronchial epithelial cells (BECs) are the primary site of RV infection, where the infection occurs both in the upper and lower respiratory epithelium equally [3]. RV can infect other cells including airway macrophages, however the virus does not replicate in these cells [4], which demonstrates the important role of BECs in the first line of defence against invading pathogens. As RVs are endocytosed into BECs viral RNAs are preferentially recognized by the RNA helicase melanoma differentiation-associated gene −5 (MDA-5, also known as IFIH1), which then signals for the induction of type I interferon (IFN-α/β) and type III IFNs (IFN-λ1/2/3). These IFNs then signal for the expression of over 300 IFN-stimulated genes (ISGs) including IFNs, CCL-5, CXCL-10, and the RNA helicases MDA-5 and retinoic acid-inducible gene–I (RIG-I or DDX58) [5]. These ISGs amplify IFN responses and induce apoptosis and limit viral replication. CCL-5 and CXCL-10 recruit cytotoxic T cells to kill virus-infected cells [5].

RV RNAs are also recognized by toll-like receptor (TLR) -3 and via nuclear factor – kappa B (NF-κB) signalling triggering the release of pro-inflammatory cytokines such as IL-6, leading to acute inflammation and fever [6]. Subjects with COPD are known to have increased inflammatory cytokines that drive increased recruitment of neutrophils due to heightened chemoattractant CXCL-8, and the neutrophils then phagocytose replicating viruses and limit viral spread [7]. However the mechanisms underlying the severe outcomes to infection in COPD and the role of BECs responses to virus infection remains unclear. Here we hypothesized that primary BECs (pBECs) from COPD subjects have an exaggerated inflammatory response and abnormal antiviral response to RV infection compared to healthy control pBECs. In this study, pBECs from subjects with COPD and healthy controls were infected RV-1B, and the immune responses to infection were assessed using whole genome microarray analysis, allowing the opportunity for discovery of novel regulators of abnormal responses in COPD.

## Methods

### Rhinovirus

RV-1B was obtained from the American Tissue Culture Collection (ATCC, USA). Virus was propagated using RD-ICAM-1 cells, clarified by centrifugation and viral titres were determined using 50% Tissue Culture Infective Dose (TCID_50_).

### Subject recruitment

Subjects with COPD were recruited; defined by a previous smoking history and fixed airflow limitation on spirometry with an FEV_1_/FVC ratio < 70%, and FEV_1_ < 80% predicted and classified by the GOLD criteria. Those with GOLD Stage III (severe COPD) FEV_1_ 30 – 50% predicted, and Stage IV (very severe COPD) FEV_1_ <30% were included. COPD subjects were ex-smokers (at least one year abstinent) and did not use inhaled corticosteroids for two weeks prior to bronchoscopy. Healthy non-smoking controls with no evidence of airflow obstruction, bronchial hyper-responsiveness to hypertonic saline challenge, or chronic respiratory symptoms were recruited. A clinical history, examination and spirometry were performed. Subjects were excluded if they had symptoms of an acute respiratory tract infection a month prior. All subjects gave written informed consent. The study was approved by The University of Newcastle Human Research Ethics.

### Cell culture and viral infection

Human pBECs were obtained by endobronchial brushing during fibre-optic bronchoscopy in accordance with standard guidelines, and cultured as described previously [8]. Cells were seeded at 1 x 10^5^ and cultured in BEBM basal media with supplements (BEGM, Lonza). At passage 2 when the cells reached 80% confluency in 24-well plates, virus infection was performed at a multiplicity of infection (MOI) of 20 for 1 hour with shaking. After 1 hour incubation viral inoculum was removed and fresh BEBM basal media supplemented with 0.5% BSA was then added. For IFN pre-treatment experiments, human recombinant IFN-β (10U) and IFN-λ1 protein (1ng/mL; R&D Systems) was used to pre-treat pBECs for 24hrs before RV-1B infection in the designated experiments. For all experiments samples were collected at 6hrs for whole genome gene expression microarray and real-time PCR (qPCR) and 24hrs for ELISA/CBA and viability/apoptosis.

### Whole genome gene expression microarray

pBEC RNA was extracted, 500ng amplified and 750ng cRNA hybridised to Illumina’s HumanRef-8 V3 BeadChips as previously described [9].

### Real-time PCR (qPCR)

RNA was extracted, reverse transcribed to cDNA and real-time PCR was performed as previously described [9]. Briefly, RNA (200ng) was reverse-transcribed to cDNA using the high capacity cDNA reverse transcription kit (Applied Biosystems, Foster City,USA). Taqman qPCR primer and probes for the target genes were purchased in kit form (Applied Biosystems, Foster City,USA). PCR primers and probes were combined with Taqman gene expression master mix as per manufacturer’s instructions in duplicate singleplex real-time PCRs (7500 Real Time PCR System, Applied Biosystems). Fold change results were calculated using 2^-ΔΔCt^ relative to the internal reference gene (18S) and the mean of all samples.

### ELISA

IFN-λ1/3, CXCL-10, IL-6, TNF-α and CCL-5 concentration was assessed using ELISA kit (IFN-λ1/3, R&D Systems, lower detection limit of 31.25pg/mL) and cytometric bead array (CXCL-10, IL-6, TNF-α and CCL-5, BD Biosciences, lower detection limit of 10pg/mL) according to the manufacturer’s instructions.

### Viability/Apoptosis

Apoptosis was measured using Annexin V-PE Apoptosis Detection Kit I (Becton Dickinson) according to manufacturer’s instructions [10]. Briefly, infected and non-infected cells were stained with Annexin V-PE and 7-AAD analysed by flow cytometry. Annexin V-PE positive and 7-AAD negative were determined to be apoptotic, Annexin V-PE and 7-AAD positive were necrotic and Annexin V-PE and 7-AAD negative were live cells.

### Viral replication

Viral replication was determined using both TCID_50_ and relative RNA copy number using a standard curve created using serial dilution of RV-1B RNA.

### Data analysis

Data analysis preformed using Stata 9 or GraphPad Prism 5 and reported as mean with standard error of means (SEM) for normally distributed data and median (quartile [Q] 1, Q3) for nonparametric data. Statistical comparisons were performed by using multiple comparisons ANOVA for parametric data and the Kruskal-Wallis test for nonparametric data. P<0.05 was considered significant.

### Whole genome gene expression microarray analysis

Microarray data were exported by using Genome Studio (Illumina) and analyzed by using GeneSpring GX11 (Agilent Technologies). Data were log-transformed, normalized, and baseline-converted to the median of all samples. Data were filtered, and only genes flagged as present (< 0.05 detection P value) in all samples were included in the further analysis. Hierarchical clustering analysis was performed by using the Pearson centered algorithm with complete linkage. The Pearson algorithms cluster samples on the basis of their correlation coefficients, whereas complete linkage measures that the distance between 2 clusters is the greatest distance between the members of the 2 clusters. Differential gene expression was determined by using ANOVA with Tukey post hoc testing (P < 0.05 adjusted for multiple comparisons by using the Benjamini-Hochberg method) and fold change >2.

## Results

### Gene expression profiles of healthy control and COPD pBECs at baseline and in response to RV-1B infection

Primary BECs from ten subjects with moderate and severe COPD and ten healthy controls were assessed *in vitro* for their immune response to RV-1B infection. Patient characteristics and details of optimisation of experimental conditions are summarised in Additional file 1: Table S1 of the online supplement. Gene expression at baseline of the non-infected media controls was first examined and showed a different expression profile between COPD and healthy pBECs (Table 1). There were 9 genes that were highly up-regulated in COPD pBECs, including genes involved in innate immunity, cell repair, and some with protease/anti-protease activity.

**Table 1:** Differential gene expression between baseline COPD pBECs and healthy control pBECs

**SYMBOL**	**GENE NAME**	**FOLD CHANGE**	**P value**
***Protease and Antiproteases***			
MMP10	Matrix metallopeptidase 10	2.94	0.0029
PI3	Peptidase inhibitor 3 (Elafin)	2.22	0.0287
ADAM19	ADAM metallopeptidase domain 19	2.13	0.0090
***Innate immunity***			
S100A8	S100 calcium binding protein A8	2.63	0.0189
S100A9	S100 calcium binding protein A9	2.32	0.0143
***Epithelial cell related processes***			
KRT6C	Keratin 6C	10.01	0.0002
RHCG	Rh family, C glycoprotein	2.51	0.0033
CNFN	Cornifelin	2.23	0.0213
SPRR2E	Small proline-rich protein 2E	2.15	0.0017

**Figure 1:**
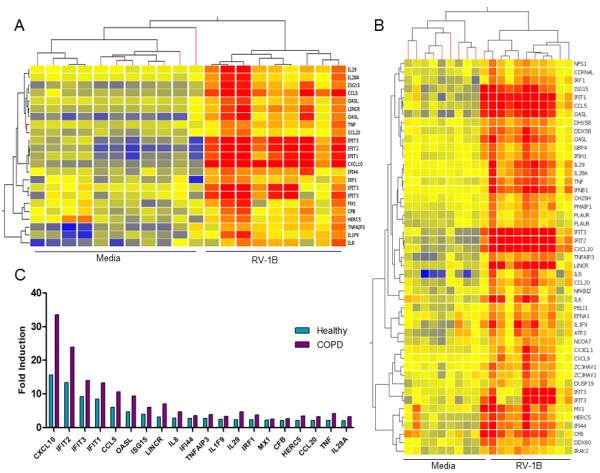
**Cluster analysis of genes significantly induced in healthy (n=10) and COPD (n=10) pBECs 6 hours after RV-1B infection.** Microarray analysis showed that RV-1B infection significantly up-regulated (**A**) 20 genes in healthy controls and (**B**) 42 genes in COPD pBECs. (**C**) All the genes that were up-regulated in both groups were significantly higher in COPD compared to that in healthy control pBECs.

Infection of pBECs with RV-1B led to changes in the gene expression profile from baseline, and differences between healthy and COPD pBEC responses were assessed. RV-1B infection caused significant up-regulation of 23 entities corresponding to 20 genes in healthy control pBECs (Figure 1A), and 48 entities corresponding to 42 genes were significantly induced in COPD pBECs (Figure 1B). Of the genes that were induced in both COPD and healthy control pBECs, all were up-regulated to higher levels in COPD compared to that in healthy pBECs (Figure 1C). There was also an additional 22 genes up-regulated in COPD pBECs in response to RV-1B infection (Table 2).

**Table 2:** Twenty two genes were increased in RV-1B infection of COPD pBECs but not changed RV-1B infection of healthy pBECs

**SYMBOL**	**GENE NAME**	**FOLD CHANGE**	**P value**	**PROBE ID**
IFNB1	Interferon, beta 1	5.8	<0.0001	1510669
IL6	Interleukin 6	3.3	0.0022	4040576
RIG-I	Retinoic acid-inducible gene – I	3.1	<0.0001	4760703
ATF3	Activating transcription factor 3	2.7	0.0007	4780128
CX3CL1	Chemokine (C-X3-C motif) ligand 1	2.5	<0.0001	630521
CXCL9	Chemokine (C-X-C motif) ligand 9	2.5	<0.0001	5570278
MDA-5	Melanoma differentiation-associated gene −5	2.5	<0.0001	4570441
ZC3HAV1	Zinc finger CCCH-type, antiviral 1	2.4	<0.0001	3710243 4540382
		2.2	<0.0001	
PMAIP1	Phorbol-12-myristate-13-acetate-induced protein 1	2.4	<0.0001	2750367
DHX58	DEXH (Asp-Glu-X-His) box polypeptide 58	2.3	<0.0001	3930681
NFS1	NFS1 nitrogen fixation 1 homolog	2.2	<0.0001	7570411
CH25H	Cholesterol 25-hydroxylase	2.2	<0.0001	1770593
CCRN4L	CCR4 carbon catabolite repression 4-like	2.2	<0.0001	460484
IRAK2	Interleukin-1 receptor-associated kinase 2	2.2	<0.0001	3930750
PELI1	Pellino homolog 1	2.2	0.0007	3450092
NCOA7	Nuclear receptor coactivator 7	2.1	0.0001	630091
PLAUR	Plasminogen activator, urokinase receptor	2.1	0.0054	730528 6220671
		2.1	0.0028	
DDX60	DEAD (Asp-Glu-Ala-Asp) box polypeptide 60	2.1	0.0001	7610053
NFKBIZ	Nuclear factor of kappa light polypeptide gene enhancer in B-cells inhibitor, zeta	2.1	0.0011	2470348
GBP4	Guanylate binding protein 4	2.1	<0.0001	1980524
EFNA1	Ephrin-A1	2.1	<0.0001	3390187
DUSP19	Dual specificity phosphatase 19	2.0	0.0019	5260195

### RV-1B infection of COPD pBECs induced higher inflammatory and antiviral responses compared to healthy controls

Microarray analysis showed that COPD pBECs induced an overall higher level of inflammatory and antiviral gene expression after RV-1B infection (Additional file 1: Table S2). The increase of selected genes detected by microarray were further validated by qPCR and confirmed by ELISA at the protein level.

**Figure 2:**
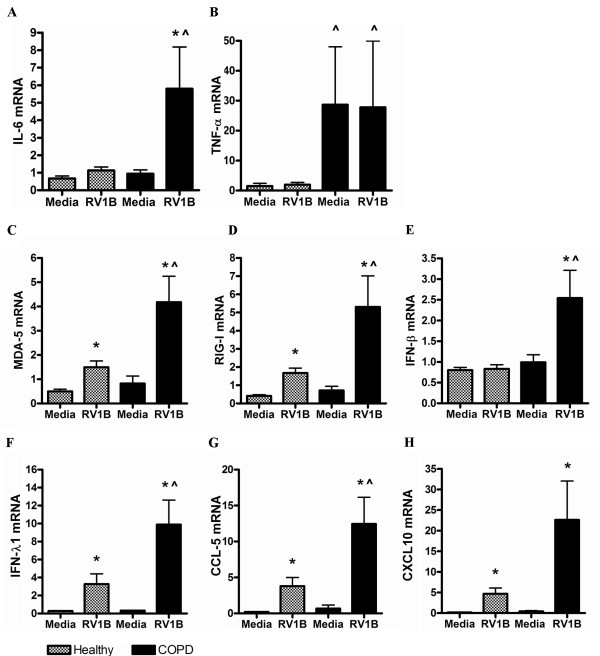
**Inflammatory and antiviral genes at 6 hours after RV-1B infection in healthy (n=10) and COPD (n=10) pBECs, confirmation of the microarray findings.** Inflammatory cytokines (**A**) IL-6 and (**B**) TNF-α were not induced by RV-1B infection in healthy control pBECs, but were IL-6 was significantly up-regulated by infection in COPD pBECs, whilst TNF-α was significantly higher in the baseline media control and after RV infection. RNA helicases (**C**) MDA-5 and (**D**) RIG-I and antiviral (**E**) IFN-β, (**F**) IFN-λ1, (**G**) CCL-5 and (**H**) CXCL-10 mRNA were all significantly higher in RV infected COPD pBECs compared to healthy controls. Results are presented as mean fold change in expression with the error bar as standard error of the mean (SEM). * p<0.05 versus the corresponding media control. ^ p<0.05 versus healthy RV infected pBECs.

RV-1B infection in healthy pBECs did not cause an up-regulation of the inflammatory genes IL-6 and TNF-α, however RV-1B infection led to a significant up-regulation of IL-6 (Figure 2A), but not TNF-α (Figure 2B) from media control in COPD pBECs. RNA helicases MDA-5 (Figure 2C) and RIG-I (Figure 2D) were up-regulated after RV-1B infection of both healthy and COPD pBECs, however the induction was significantly higher in the COPD group. Induction of IFN-λ1, and ISGs CCL-5 and CXCL-10 was significantly higher in COPD pBECs after infection compared to that in healthy pBECs (Figure 2F – H). IFN-β was only induced in COPD pBECs after infection (Figure 2E).

**Figure 3:**
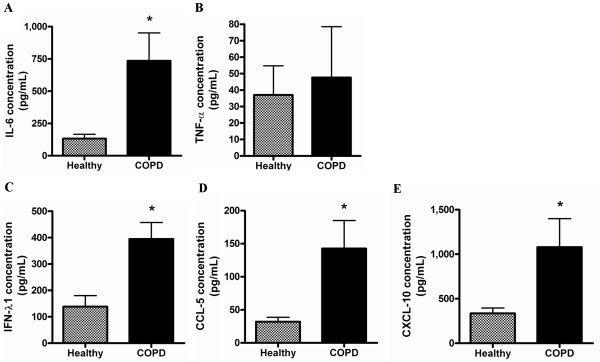
**Inflammatory and antiviral proteins at 24 hours after RV-1B infection in healthy (n=10) and COPD (n=10) pBECs.** (**A**) IL-6 protein induction after infection was significantly higher in COPD pBECs however (**B**) TNF-α induction was not significantly different between healthy and COPD pBECs. (**C**) IFN-λ1, (**D**) CCL-5 and (**E**) CXCL-10 protein induction in COPD pBECs was also significantly higher than that in healthy pBECs. Results are presented as mean and the error bar as standard error of the mean (SEM). * p<0.05 versus healthy RV infected pBECs.

All proteins were induced above media control and no difference was observed at this baseline level between healthy and COPD pBECs (data not shown). Both healthy and COPD pBECs induced significant levels of IL-6 and TNF-α at 24hr after RV-1B infection (Figure 3A – B), with IL-6 being significantly higher in COPD pBECs. Similarly IFN-λ1, CCL-5, and CXCL-10 protein was also significantly higher in COPD than in healthy pBECs after RV-1B infection (Figure 3C – E).

Surprisingly the high inflammatory and antiviral induction in COPD pBECs did not affect viral replication (Additional file 1: Figure S1 of the online supplement), as viral RNA and viral titre at 24hr was similar in healthy and COPD pBECs. RV-1B infection in both healthy and COPD pBECs resulted in a significant decrease in cell viability (Additional file 1: Figure S2A of the online supplement), however there was more apoptosis in the RV infected COPD pBECs (Additional file 1: Figure S2B and E2C of the online supplement).

### The heightened inflammatory response in COPD pBECs is due to enhanced immune signalling caused by RV-1B infection

**Figure 4:**
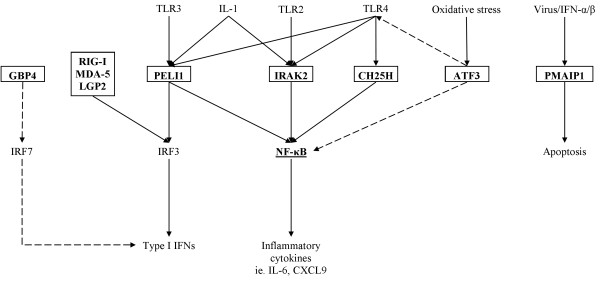
**Key signalling molecules and pathways involved in RV induced inflammatory responses in COPD pBECs.** Genes that were identified to play a role in innate immune signalling that were increased in RV infected COPD pBECs include IRAK2, PELI1, CH25H, PMAIP1, ATF3 and GBP4. IRAK2, PELI1 and CH25H (bolded in box) are involved in signal transduction downstream of IL-1/TLR leading to NF-κB activation and production of inflammatory cytokines. Induction of PMAIP1, a mitochondria-associated protein, leads to apoptosis. ATF3 senses cellular stress and functions to reduce TLR4-mediated NF-κB signalling. GBP4 is an IFN-inducible GTPase that has been shown to disrupt IRF7 activation, thereby inhibiting the induction of type I IFNs. Solid line indicates up-regulation, and dashed line indicates down-regulation.

In order to understand the mechanisms underlying the increased immune responses in COPD pBECs, we further examined the genes that are involved in immune signalling and that were up-regulated greater than 2 fold by RV-1B infection in COPD but not healthy pBECs (Table 2). These genes were reviewed for their role in innate immunity in published literature (Pubmed), and those with known functions were mapped into the currently known intracellular innate immune signalling pathways. Figure 4 illustrates the key novel innate immune signalling genes involved in RV induced inflammatory responses in COPD pBECs. Signalling via IL-1 and TLR pathways was apparent, involving Pellino 1 (PELI1), IL-1 receptor-associated kinase-like 2 (IRAK2) and cholesterol 25-hydroxylase (CH25H). Oxidative stress appears important relating to the up-regulation of activating transcription factor 3 (ATF3) and guanylate binding protein 4 (GBP4), and phorbol-12-myristate-13-acetate-induced protein 1 (PMAIP1 or Noxa) plays a role in apoptosis (Figure 4).

### Pre-treatment with IFN-β and IFN-λ1 led to enhanced antiviral responses to RV-1B infection in both healthy and COPD pBECs

**Figure 5:**
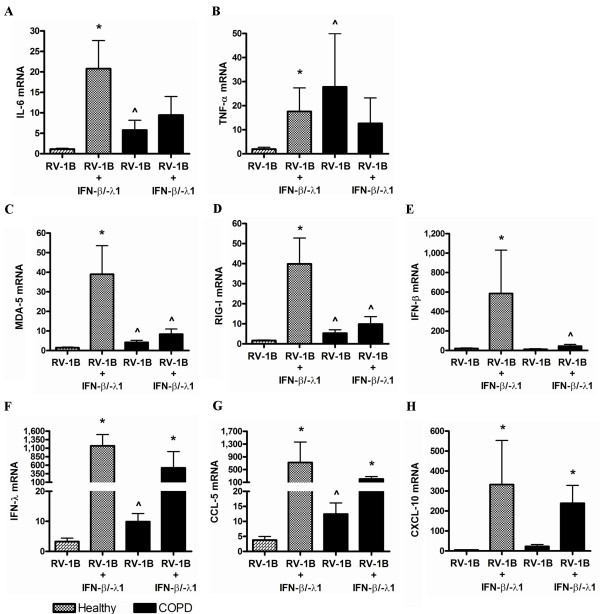
**Inflammatory and antiviral genes at 6 hours after RV-1B infection in IFN-β/λ1-pre-treated healthy (n=6) and COPD (n=6) pBECs.** IFN-β/λ1-pre-treatment before RV-1B infection resulted in a significant increase in (**A**) IL-6 and (**B**) TNF-α mRNA in healthy but not in COPD pBECs. (**C**) MDA-5, (**D**) RIG-I, and (**E**) IFN-β mRNA was significantly induced and higher in healthy control than in COPD pBECs. In sharp contrast, (**F**) IFN-λ1, (**G**) CCL-5, and (**H**) CXCL-10 mRNA was significantly up-regulated by IFNs pre-treatment in both healthy and COPD pBECs. Results were presented as mean fold change in expression with and the error bar as standard error of the mean (SEM). * p<0.05 versus the corresponding RV-1B alone. ^p<0.05 versus healthy RV-1B infected pBECs.

**Figure 6:**
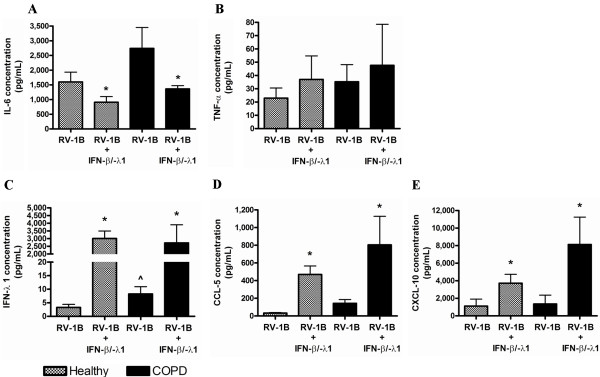
**Inflammatory and antiviral proteins at 24 hours after RV-1B infection in IFN-β/λ1-pre-treated healthy (n=6) and COPD (n=6) pBECs.** IFN-β/λ1-pre-treatment before RV-1B infection led to a reduction of (**A**) IL-6 in healthy pBECs, but had no effect in COPD pBECs. (**B**) Similarly TNF-α protein was not affected by IFNs pre-treatment. (**C**) IFN-λ1, (**D**) CCL-5, and (**E**) CXCL-10 was significantly induced in pre-treated healthy and COPD pBECs. Results were presented as standard error of the mean (SEM). * p<0.05 versus the corresponding RV-1B. ^p<0.05 versus healthy RV-1B infected pBECs.

COPD pBECs produced a greater antiviral response to RV infection, but this was not associated with reduced viral replication. We sought to determine if IFN-β/λ1 pre-treatment affects the inflammatory and antiviral responses to RV-1B infection. pBECs were primed with a combination of exogenous IFN-β and IFN-λ1 before infection. IFN-β/λ1 pre-treatment led to a significant induction of IL-6 and TNF-α mRNA in healthy control but had no effect in COPD pBECs (Figure 5A – B). While the RNA helicases and IFN-β mRNA was significantly up-regulated in healthy control pBECs by the pre-treatment, these genes were not induced in COPD pBECs following pre-treatment (Figure 5C – E). IFN-λ1, CCL-5, and CXCL-10 mRNA however were significantly induced in both pBECs groups after IFNs pre-treatment (Figure 5F – H). Surprisingly release of IL-6 and TNF-α protein did not increase with the up-regulated gene expression. IL-6 protein was in fact reduced and TNF-α remained unaffected following IFN-β/λ1 pre-treatment (Figure 6A – B). However, IFN-λ1, CCL-5, and CXCL-10 proteins were significantly induced in both healthy and COPD pBECs after pre-treatment (Figure 6C – E). The heightened antiviral responses also correlated with significantly reduced viral titre with exogenous IFN-β/λ1 treatment (Additional file 1: Figure S3 of the online supplement).

## Discussion

People with COPD are more susceptible to viral infection and suffer severe complications with worsened symptoms and frequent exacerbations following infection. This study investigated the transcriptional response of pBECs to RV infection and how this is altered in COPD. We show that the immune response of healthy and COPD cells was characterised by a robust up-regulation of pro-inflammatory and antiviral pathways. However there were clear differences between the COPD and healthy pBECs, including up-regulation of inflammatory genes at baseline and dramatically exaggerated responses to RV infection. We identified 9 genes associated with COPD at baseline and 22 genes altered in COPD but not healthy pBECs in response to RV, not previously reported in COPD, but likely to be important in regulating the exaggerated virus induced inflammation. The increased gene expression in RV infected COPD pBECs also correlated with the corresponding protein release, and enhanced apoptosis; however this enhanced immune response did not reduce viral titre. Furthermore, IFN-β/λ1 pre-treatment resulted in enhanced responses to RV-1B in COPD and healthy pBECs, however COPD pBECs were unresponsive in terms of MDA-5/RIG-I and IFN-β gene induction. Despite this abnormality IFN-β/λ1 pre-treatment still led to significantly reduced viral titre.

The airway epithelium in COPD is exposed chronically to enhanced airway inflammation with increased numbers of neutrophils and lymphocytes that correlate with more severe airflow obstruction, despite the fact that the majority of subjects have ceased smoking, indicating that the airway inflammatory response in COPD becomes self-perpetuating [11,12]. We found several novel associations with 9 up-regulated genes in COPD pBECs, including the calgranulins S100A8 and S100A9, and proteases ADAM19 and MMP10. S100A8/A9 are small calcium-binding proteins with pro-inflammatory activity and have been reported to increase with steroid resistant neutrophilic inflammation [13]. ADAM19 and MMP10 are involved in tissue repair and remodelling, and single nucleotide polymorphisms in the gene locus containing ADAM19 has been associated with COPD [14]. These observations are consistent with other studies suggesting that the airways of COPD subjects undergo constant cellular repair due to the damages caused by cigarette smoke exposure [1]. Interestingly MMP10 and ADAM19 gene expression has also been shown to be upregulated in pBECs from subjects with asthma [15].

Upon RV-1B infection, both pBEC groups mounted a robust inflammatory response; however the response was more exaggerated in COPD pBECs. This was in accordance with other studies that showed a more vigorous inflammatory response in COPD pBECs against RV infection [16]. We have identified a number of novel genes whose expression were strongly up-regulated in COPD pBECs in response to RV-1B but not in healthy pBECs. These include important signalling molecules downstream of IL-1 and TLR2, 3 and 4 pathways such as PELI1 [17], IRAK2 [18] and CH25H [19]. We have previously shown that PELI1 and IRAK2 are up-regulated in the sputum of subjects with neutrophilic asthma [9]. This suggests that IRAK2 and PELI1 play a role promoting neutrophilic airway inflammation, which is triggered by RV infection of the epithelium in COPD. Recently, PELI1 has been shown to be important in regulating the innate immune response of the epithelium to RV, including CXCL-8 production and neutrophil recruitment, without interfering with IFN responses and viral replication [20]. Mouse models have shown that IRAK2 is critical in sustaining late phase inflammatory responses after TLR stimulation, leading to increased production of inflammatory cytokines [21]. These molecules may also be important in T cell related functions, such as T cell tolerance [22], and promotion of Th17 cell development [23].

Other important signalling proteins induced by RV in COPD pBECs include PMAIP1, ATF3 and GBP4. PMAIP1, a mitochondria-associated protein, promotes apoptosis [24]. ATF3 senses oxidative stress [25] and functions to reduce TLR4-mediated NF-κB signalling, therefore serving as negative feedback [26]. GBP4 is an IFN-inducible GTPase that has been shown to disrupt IRF7 activation, thereby inhibiting the induction of type I IFNs [27]. Also potentially important is the up-regulation of Chemokine (C-X3-C motif) ligand 1 (CX3CL1), more commonly known as fractalkine, by RV-1B in COPD pBECs. Soluble CX3CL1 has a chemo-attractant activity for T cells and monocytes; whereas membrane bound protein promotes adhesion of these leukocytes [28].

Antiviral responses such as IFN-β and -λ1 are critical in limiting viral replication, and were significantly higher in COPD pBECs. This was associated with the higher level of apoptosis after infection, but surprisingly did not affect viral titre in COPD, which was similar to the healthy group. This was in contrast to the study by Schneider et al. that showed enhanced antiviral responses associated with increased replication of RV-39 after infection in COPD [29]. Other studies have examined transcriptional responses to *in vivo* RV infection of healthy controls using nasal epithelial scrapings [30], or *in vitro* RV infection of healthy pBECs [31]. There are many similarities between the changes in gene expression found in reported studies compared to the current study, which includes upregulation of ISGs, antiviral genes, chemokines, and cytokines. However there are also differences in the healthy pBEC responses, including some genes previously reported to be upregulated by RV (e.g. IFNB1, and IL6) that were unchanged in this study. These differences are likely explained by altered experimental conditions, such as differing strains of RV, culture or sampling methods including submerged culture versus air-liquid interface culture versus *in vitro* infection, time points of RNA sampling, microarray platforms and microarray analysis methods. Nevertheless, this well controlled study adds significant knowledge regarding the differences between healthy and COPD innate immune responses to RV-1B infection under the conditions investigated, which warrant further investigation.

The underlying mechanisms of unchanged viral replication despite induced antiviral responses in COPD pBECs after RV-1B infection is currently unclear. It is possible that the IFNs produced by infection did not efficiently initiate the subsequent inductions of ISGs in COPD pBECs, therefore leading to an unchanged viral titre. However this would not explain the high apoptosis induction in COPD pBECs by RV-1B infection and marked reduction of RV-1B viral replication following IFN pre-treatment. ISGs such as protein kinase R (PKR) have been shown to induce apoptosis via the Fas-associated death domain (FADD) [32], and induction of which by RV-1B infection in COPD pBECs was higher compared to that in healthy pBECs. Pre-treatment with IFNs also significantly reduced viral replication, further suggesting IFN signalling leading to ISG induction may be functional in COPD. Nevertheless, it is also possible that ISGs were ineffectively up-regulated in COPD pBECs, and apoptosis could also have been induced by other signalling pathways including TNF-α/TNFR1 pathway [33], and compensated for the reduced ISGs production. This may explain the excess tissue damage and high inflammation that can be caused by high levels of apoptosis in the airways of those with COPD [34].

While we showed a heightened inflammatory response and antiviral response to RV infection in COPD pBECs in this study, we also demonstrated defects in the antiviral pathway. IFN-β/λ1 pre-treatment led to increased IL-6 and TNF-α mRNA in healthy pBECs, however this was not translated to protein production which was either reduced or unchanged with IFN-β/λ1 pre-treatment. Previous studies have shown exogenous IFN-β decreases RV-1B-induced IL-6 release from healthy and asthmatic pBECs via an unidentified pathway [35]. IFN-β/λ1 pre-treatment also enhanced antiviral responses including MDA-5 and RIG-I, and IFN-β mRNAs in healthy pBECs, leading to marked decrease in viral titre. However, in COPD pBECs IFNs pre-treatment failed to induce MDA-5 and RIG-I and IFN-β mRNA, but did enhance CCL-5, CXCL-10, and IFN-λ1 production, resulting in a decreased viral titre. This indicates that MDA-5-initiated antiviral responses were partially impaired in COPD and led to reduced IFN-β level. It is possible that IFN-β/λ1 pre-treatment significantly up-regulated ISGs such as PKR and MxA, that bound and degraded viral RNAs as RV endocytosed into the host cells. This also suggests differential signalling pathways that regulate type III IFNs other than MDA-5/RIG-I and IFN-β. Indeed, a recent study has identified a cluster of NF-κB binding sites on human IFN-λ1 promoter, and NF-κB was critical for IFN-λ1 induction but not for type I IFNs expression [36]. This is consistent with our results as both NF-κB and IFN-λ1were significantly up-regulated in COPD pBECs.

Apoptosis is another important component of antiviral responses, which was significantly increased in COPD pBECs after RV infection. This may correlate with the increased TNF and IFN levels [37], which can also up-regulate PMAIP1 gene that promotes the induction of apoptosis [24]. However increased apoptosis did not reduce viral titre. The reason for this observation is unclear; however, it is possible that the quantification methods (TCID_50_) used in this study may not be as sensitive as direct quantification methods such as plaque assays. Detection of RV-1B by qPCR could only measure the level of total viral RNAs and not differentiate live from dead viruses. Alternatively, high levels of oxidative stress could have contributed to this observation. Superoxide dismutase 2 (SOD2) and ATF3 are important anti-oxidative genes that were up-regulated to a greater extent in COPD pBECs after infection when compared with infected healthy pBECs. Lack of SOD2 in mice can lead to increased oxidative damage to DNA [38], and lack of ATF3 alters DNA repair mechanisms [39]. This indirectly indicates that the level of oxidative stress is higher in RV infected COPD pBECs compared to that in healthy cells.

## Conclusions

We have demonstrated that COPD pBECs elicit excessive pro-inflammatory and antiviral responses to RV-1B infection. The greater expression of molecules such as the calgranulins at baseline and pellino-1 and IRAK2 after RV-1B infection may contribute to dysregulated innate immune responses in the airways and potentiation of inflammation as seen in COPD. Additionally, MDA-5/RIG-I and IFN-β induction to exogenous IFN-β/λ1 pre-treatment prior to infection was impaired in COPD. However IFN-λ1 induction by RV-1B infection was sufficient to limit replication in COPD pBECs. This provides novel insight in the immune responses in COPD pBECs to RV infection and may reveal potential therapeutic targets that limit the dysregulated inflammatory response due to RV infection, without compromising antiviral defences in COPD.

## Abbreviations

RV: Rhinovirus; COPD: Chronic obstructive pulmonary disease; pBEC: Primary bronchial epithelial cell; qPCR: Quantitative real-time polymerase chain reaction; ELISA: Enzyme linked immunosorbent assay; RNA: Ribonucleic acid; IFN: Interferon; MDA-5: Melanoma differentiation-associated gene −5; RIG-I: Retinoic acid-inducible gene–I; CXCL-10: Chemokine (C-X-C) motif ligand 10; CCL-5: Chemokine (C-C motif) ligand 5; TLR: Toll-like receptor; NF-κB: Nuclear factor-κB; IL: Interleukin; CXCL8: Chemokine (C-X-C) motif ligand 8; FEV_1_: Forced expiratory volume exhaled in 1 second; GOLD: Global initiative for chronic obstructive lung disease; MOI: Multiplicity of infection; Ct: Cycle threshold; TNF: Tumor necrosis factor; SEM: Standard error of means; ANOVA:Analysis of variance.

## Competing interests

The authors declare they have no competing interests.

## Authors’ contributions

KJB, PGG, and PABW participated in the study’s conception and design. PABW performed subject recruitment. KJB, MT and LPG performed the experiments. ACYH and KJB performed statistical analysis and all authors participated in the interpretation of data, preparation and editing of manuscript for intellectual content. All authors read and approved the final manuscript.

## Authors’ information

KJ Baines and AC-Y Hsu are joint first authors to this manuscript.

## Supplementary Material

Additional file 1 **Online Supplementary Material. Table S1.** Subject characteristics. FEV_1_% predicted refers to the forced expiratory volume in 1s expressed as a percentage of the predicated value. **Table S2.** Differential gene expression of RV-1B infected COPD pBECs compared to RV-1B infected healthy control pBECs**. Figure S1.** RV-1B viral replication in healthy and COPD pBECs at 24hr after infection. RV-1B viral replication was measured by (A) qPCR and by (B) TCID_50_ assay, and viral replication was similarly observed in both healthy and COPD pBECs after infection. Results were presented as standard error of the mean (SEM). **Figure S2.** Viability, apoptosis, and necrosis induction after RV-1B infection. Host cellular viability, apoptosis, and necrosis was determined in the infected pBECs. (A) Healthy and COPD pBECs showed a significant reduction in viability after infection, and this correlated with concomitant increase in (B) apoptosis and (C) necrosis. Results were presented as standard error of the mean (SEM). * indicates a significant difference compared to the relative media control. ^ indicates a significant difference compared to healthy pBECs media control. **Figure S3.** RV-1B viral replication in IFN-β/λ1-pre-treated pBECs. (A) Viral RNAs was not affected at RNA levels, however (B) viral replication by TCID_50_ assay showed a decrease in replication with IFN-β/λ1 treatment. Results were presented as standard error of the mean (SEM). * indicates a significant difference compared to the relative media control. Click here for file
