# Supplementary material for: Novel immune genes associated with excessive inflammatory and antiviral responses to rhinovirus in COPD
Source: Respir Res. 2013 Feb 6;14(1):15. doi: 10.1186/1465-9921-14-15 (PMC3570361; doi:10.1186/1465-9921-14-15)
Supplement: Additional file 1 — Online Supplementary Material. Table S1. Subject characteristics. FEV1% predicted refers to the forced expiratory volume in 1s expressed as a percentage of the predicated value. Table S2. Differential gene expression of RV-1B infected COPD pBECs compared to RV-1B infected healthy control pBECs. Figure S1. RV-1B viral replication in healthy and COPD pBECs at 24hr after infection. RV-1B viral replication was measured by (A) qPCR and by (B) TCID50 assay, and viral replication was similarly observed in both healthy and COPD pBECs after infection. Results were presented as standard error of the mean (SEM). Figure S2. Viability, apoptosis, and necrosis induction after RV-1B infection. Host cellular viability, apoptosis, and necrosis was determined in the infected pBECs. (A) Healthy and COPD pBECs showed a significant reduction in viability after infection, and this correlated with concomitant increase in (B) apoptosis and (C) necrosis. Results were presented as standard error of the mean (SEM). * indicates a significant difference compared to the relative media control. ^ indicates a significant difference compared to healthy pBECs media control. Figure S3. RV-1B viral replication in IFN-β/λ1-pre-treated pBECs. (A) Viral RNAs was not affected at RNA levels, however (B) viral replication by TCID50 assay showed a decrease in replication with IFN-β/λ1 treatment. Results were presented as standard error of the mean (SEM). * indicates a significant difference compared to the relative media control. [file 1465-9921-14-15-S1.doc]

# Novel immune genes associated with excessive inflammatory and antiviral responses to rhinovirus in COPD

Katherine J. Baines^1,2*^

Email: [katherine.baines@newcastle.edu.au](mailto:katherine.baines@newcastle.edu.au)

Alan C-Y. Hsu^1,2*^

Email: [alan.hsu@newcastle.edu.au](mailto:alan.hsu@newcastle.edu.au)

Melinda Tooze^1,2^

Email: [melinda.tooze@newcastle.edu.au](mailto:melinda.tooze@newcastle.edu.au)

Lakshitha P. Gunawardhana^1,2^

Email: [lakshitha.gunawardhana@newcastle.edu.au](mailto:lakshitha.gunawardhana@newcastle.edu.au)

Peter G. Gibson^1,2,3^

Email: [peter.gibson@hnehealth.nsw.gov.au](mailto:peter.gibson@hnehealth.nsw.gov.au)

Peter AB. Wark^1,2,3^

Email: [peter.wark@hnehealth.nsw.gov.au](mailto:peter.wark@hnehealth.nsw.gov.au)

1. Priority Research Centre for Asthma and Respiratory Disease, The University of Newcastle, Callaghan, NSW, AUSTRALIA
2. Respiratory Medicine, Hunter Medical Research Institute, New Lambton Heights, NSW, AUSTRALIA
3. Department of Respiratory and Sleep Medicine, John Hunter Hospital, New Lambton Heights, NSW, AUSTRALIA

*KJ Baines and AC-Y Hsu are joint first authors and contributed equally to this manuscript.

Corresponding Author Details: Dr Katherine Baines

Level 2 West Wing, Hunter Medical Research Institute

Lot 1, Kookaburra Circuit

New Lambton Heights, NSW 2305, AUSTRALIA

Email: katherine.baines@newcastle.edu.au

Phone: +61 2 40420090

# Online Supplementary Material

# Results

**Experiment Optimisation**

Healthy and COPD pBECs were then infected with RV-1B for 24hr and gene expression was then profiled. A dose- and time-response of RV-1B was determined on pBECs, and an MOI of 20 was shown to elicit appropriate induction of immune responses without causing significant cytopathic effect. Thus an MOI of 20 was used in all experiments. UV-inactivated RV-1B was also used as negative control.

**Table S1**: Subject characteristics

|  | **Healthy** | **COPD** | **P - value** |
| --- | --- | --- | --- |
| Number | 10 | 10 | NA |
| Sex (percent female) | 40% | 60% | P = 0.6 |
| Age, mean (SD) | 47 (44) | 66 (26) | P = 1.0 |
| FEV_1_ % predicted, mean | 96.3% | 36.1% | P < 0.001 |
| Smoking history, mean pack years | 0 | 54 | NA |
| Years since quit smoking, mean | 0 | 11.25 | NA |
| ICS (%) | 0 | 80% | NA |

FEV_1_% predicted refers to the forced expiratory volume in 1s expressed as a percentage of the predicated value.

**Table S2: Differential gene expression of RV-1B infected COPD pBECs compared to RV-1B infected healthy control pBECs**

| **SYMBOL** | **GENE NAME** | **FOLD CHANGE** | **P value** |
| --- | --- | --- | --- |
| ***IFN and IFN stimulated genes*** | |  |  |
| IFNB1 | Interferon, beta 1 | 3.08 | <0.0001 |
| IL29 | Interleukin 29 (interferon, lambda 1) | 2.03 | <0.0001 |
| IFIT1 | Interferon-induced protein with tetratricopeptide repeats 1 | 2.65 | <0.0001 |
| IFIT2 | Interferon-induced protein with tetratricopeptide repeats 2 | 2.72 | <0.0001 |
| IFIT3 | Interferon-induced protein with tetratricopeptide repeats 3 | 2.32 | <0.0001 |
| ISG15 | ISG15 ubiquitin-like modifier | 2.34 | <0.0001 |
| MX1 | Myxovirus (influenza virus) resistance 1, interferon-inducible protein p78 | 2.19 | 0.0007 |
| OASL | 2'-5'-oligoadenylate synthetase-like | 3.13 | <0.0001 |
| ***Chemokines and Cytokines*** | |  |  |
| CXCL10 | Chemokine (C-X-C motif) ligand 10 | 3.07 | <0.0001 |
| CCL5 | Chemokine (C-C motif) ligand 5 | 2.56 | <0.0001 |
| TNF | Tumor necrosis factor (TNF superfamily, member 2) | 2.18 | <0.0001 |
| IL1F9 | Interleukin 1 family, member 9 | 2.06 | <0.0001 |
| ***Innate immunity*** | |  |  |
| CFB | Complement factor B | 2.45 | 0.0006 |
| SERPINB4 | Serpin peptidase inhibitor, clade B (ovalbumin), member 4 | 2.03 | 0.0406 |
| EDN1 | Endothelin 1 | 2.11 | 0.0094 |
| ***Oxidative stress related genes*** | |  |  |
| SOD2 | Superoxide dismutase 2, mitochondrial | 2.06 | <0.0001 |
| HMOX1 | Heme oxygenase (decycling) 1 | 2.02 | 0.0005 |


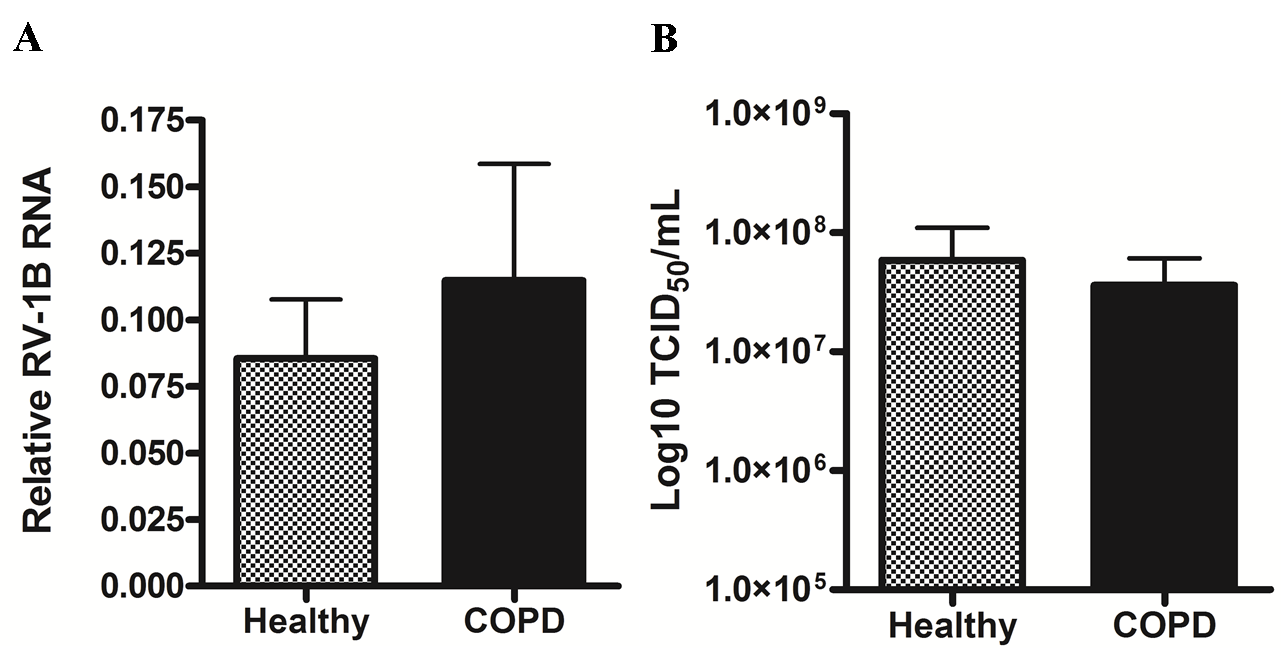


**Figure S1. RV-1B viral replication in healthy and COPD pBECs at 24hr after infection.** RV-1B viral replication was measured by (A) qPCR and by (B) TCID_50_ assay, and viral replication was similarly observed in both healthy and COPD pBECs after infection. Results were presented as standard error of the mean (SEM).


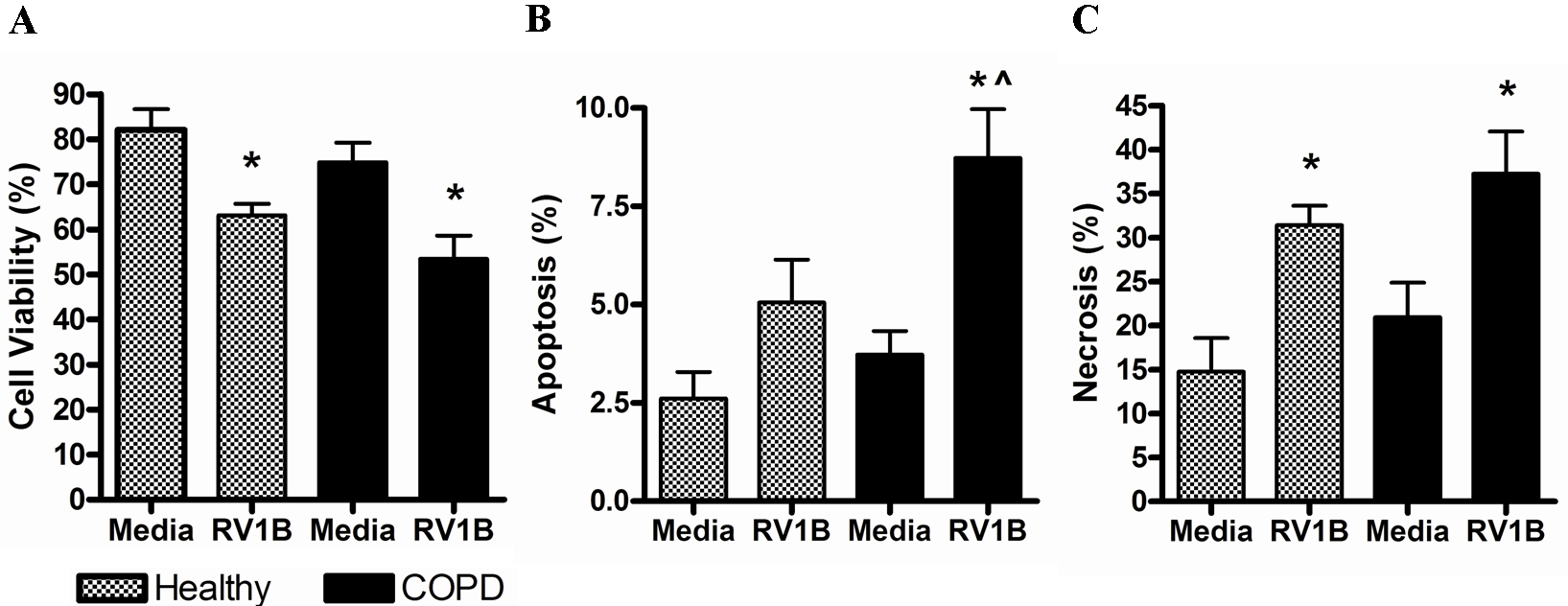


**Figure S2. Viability, apoptosis, and necrosis induction after RV-1B infection.** Host cellular viability, apoptosis, and necrosis was determined in the infected pBECs. (A) Healthy and COPD pBECs showed a significant reduction in viability after infection, and this correlated with concomitant increase in (B) apoptosis and (C) necrosis. Results were presented as standard error of the mean (SEM). * indicates a significant difference compared to the relative media control. ^ indicates a significant difference compared to healthy pBECs media control.


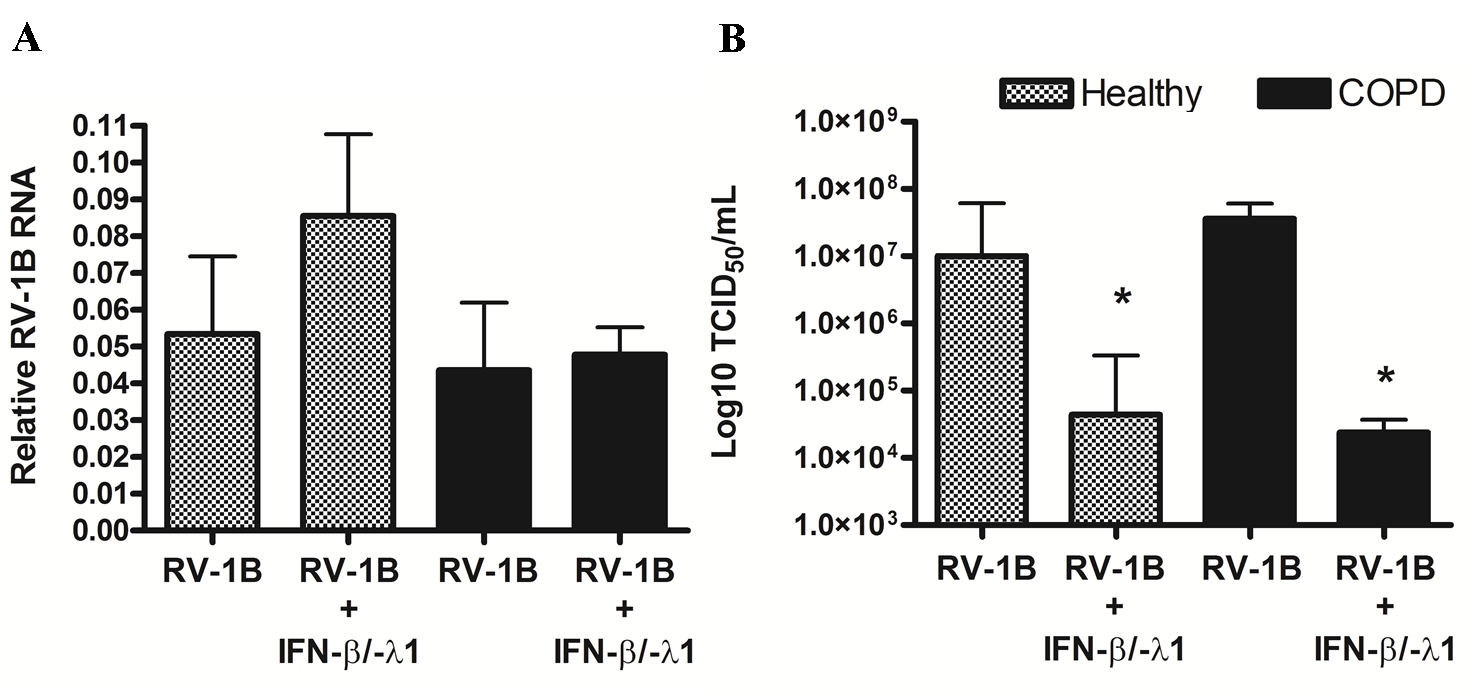


**Figure S3. RV-1B viral replication in IFN-β/λ1-pre-treated pBECs.** (A) Viral RNAs was not affected at RNA levels, however (B) viral replication by TCID_50_ assay showed a decrease in replication with IFN-β/λ1 treatment. Results were presented as standard error of the mean (SEM). * indicates a significant difference compared to the relative media control.
